# Sociodemographics, behaviour and knowledge of first South African HPV-vaccine recipients

**DOI:** 10.4102/safp.v66i1.5913

**Published:** 2024-04-24

**Authors:** Robyn A. Adams, Cathy Visser, Greta Dreyer, Leon Snyman, Frederick H. van der Merwe, Matthys H. Botha

**Affiliations:** 1 Department of Obstetrics and Gynecology, Faculty of Medicine and Health Science, Stellenbosch University, Cape Town, South Africa; 2 Department of Obstetrics and Gynecology, Faculty of Medicine and Health Science, University of Pretoria, Pretoria, South Africa

**Keywords:** human papillomavirus vaccine, cervical cancer, sexual practices, education, cervical cancer prevention

## Abstract

**Background:**

Infection with the human papillomavirus (HPV) is a necessary cause of cervical cancer and is one of the most prevalent sexually transmitted infections worldwide. Primary prevention strategies target reducing HPV acquisition through vaccination, limiting exposure (e.g. delayed sexual debut, barrier contraception) and health education focusing on sexual behaviour and tobacco use.

**Methods:**

The ImmunoVACCS study, conducted from 2019 to 2022 in two provinces in South Africa, examined sociodemographic characteristics, sexual practices, and knowledge of cervical cancer and the HPV vaccine among young female vaccine recipients. It encompassed participants from the previously conducted vaccine implementation trials, VACCS 1 and VACCS 2 (2011–2014). Recruitment involved telephonic contact with eligible potential participants. Data were collected through self-administered questionnaires.

**Results:**

One hundred and eleven participants took part in the current study (median age: 20 years; age range: 16–22 years). Most sexually active participants had their first engagement in secondary school (96.2%), and 77.2% used contraception during their last sexual activity. Knowledge gaps were evident, with only 13.5% recognising cervical cancer’s cervix origin and 3.6% attributing it to a virus. Despite this, 70.3% had heard of a vaccine for cervical cancer. Less than half knew about the importance of regular Pap smears (49.5%), vaccine protection (44.1%) or condom use (20.7%) against HPV and cervical cancer.

**Conclusion:**

The current study demonstrates that young women still lack complete information on cervical cancer and its risk factors even after receiving health education linked with vaccination.

**Contribution:**

This study underscores the necessity of ongoing education about HPV, its risks and preventive measures among young women to combat cervical cancer.

## Background

Infection with the human papillomavirus (HPV) is a necessary cause of cervical cancer and is one of the most prevalent sexually transmitted infections worldwide.^1^ The primary prevention of cervical cancer aims to reduce the risk of HPV acquisition. This may include vaccination against HPV, reduction of exposure to HPV (delayed sexual debut or barrier contraception), or health education focused on sexual behaviour and tobacco use.^2^ At present, there are three prophylactic HPV vaccines registered and commercially available in South Africa: the bivalent vaccine Cervarix^®^ (GlaxoSmithKline), containing virus-like particles (VLPs) for HPV 16 and HPV 18; the quadrivalent vaccine Gardasil^®^ (Merck Inc), containing VLPs for HPV 6, HPV 11, HPV 16 and HPV 18 and the nonavalent vaccine Gardasil^®^9 (Merck Inc), which provides additional protection against HPV types 31, 33, 45, 52 and 58.

The HPV vaccine is most effective before exposure to HPV and has thus been recommended for routine vaccination between the ages of 9 and 12 years.^3,4,5^ The South African HPV vaccination programme was initiated in 2014, through the Integrated School Health Programme with the aim of administering the bivalent, two-dose schedule vaccine to girls aged 9–12 years, 5–6 months apart.^6,7^

Human papillomavirus vaccines are safe and exhibit sustained efficacy, effectively preventing the occurrence of new HPV infections.^8,9^ Despite this, vaccine coverage remains low. In 2022, data from South Africa indicated that only 47% of the targeted population had received both the initial and final doses of the vaccine. While this figure remains a concern, it marks a notable improvement from the coverage rates seen in 2020, during the height of the COVID-19 pandemic, when only 3% had received the first dose and none had completed the vaccination regimen.^10^ This upward trend suggests some success in endeavours to broaden vaccine accessibility and acceptance. Nonetheless, sustaining and enhancing these efforts are imperative to achieve higher coverage rates.

Barriers associated with HPV vaccination, as described by Kutz et al. in a 2023 systematic review, include limited health system capacities, socioeconomic status, stigma, fear and costs of vaccines, lack of correct information and health education, among others.^11^ Similarly, Wigle et al. in a 2013 systematic review focused on HPV vaccine implementation in low- and middle-income countries (LMICs) and identified three primary barriers: sociocultural, health system and political. Sociocultural included low knowledge of HPV and its relation to cervical cancer and societal values and stigma, among others.^12^

The current publication reports on the follow-up of participants previously enrolled in the VACCS 1^13,14,15^ and VACCS 2^14,16^ trials. Briefly, two cross-sectional observation trials were conducted to study different approaches to the potential linkage between HPV vaccination, education and screening. The primary objective of these studies was to evaluate whether HPV vaccine implementation can be linked successfully with other health interventions to improve maternal cervical cancer knowledge and screening in the Gauteng and Western Cape provinces of South Africa. Twenty-nine primary schools in low socioeconomic areas in the Western Cape and Gauteng provinces were identified and contacted to invite them to participate in these prospective demonstration studies.

The aim of the current study is to describe the typical female vaccine recipients’ sociodemographic characteristics, self-reported sexual behaviour, knowledge of cervical cancer and the HPV vaccine, to understand risks and protection. Antibody levels and HPV type-specific infection will be reported in a separate publication.

## Materials and methods

The present research forms a component of the ImmunoVACCS (immunity, knowledge, behaviour and HPV infection rates after preadolescent HPV vaccination during the VACCS 1 and VACCS 2 trials) study, conducted from 2019 to 2022 in the Gauteng and Western Cape provinces of South Africa.

The study population included girls invited for HPV vaccination during both the VACCS 1 and VACCS 2 trials. Potential participants were contacted telephonically by trained nurse investigators or research assistants, using the documented mobile phone numbers obtained during the VACCS 1 and VACCS 2 trials. Participants who expressed interest in participating in the current study were given a study visit at which consent for participation was obtained. During the study visit, participants aged 18 years and older provided written informed consent, while parental consent and participant assent were obtained for participants younger than 18 years.

### Data collection

A traditional pen and paper questionnaire was developed from published studies available in the public domain,^17,18^ adapted for local settings through experts and validated in a small pilot study. This self-administered anonymous questionnaire contained questions on sociodemographic characteristics, self-reported sexual behaviour, contraceptive behaviour and knowledge of cervical cancer and prevention. Each participant completed the anonymous questionnaire in a private area during the study visit but was allowed clarification of questions from the researcher. Upon completion, questionnaires were collected in a mailbox-type container.

### Data management

Microsoft Excel^®^ was used as primary software for data recording, cleaning and analysis. Checkbox replies were captured in coded format on a Microsoft Excel^®^ template. One question on the participant’s understanding of cervical cancer prompted an open-ended answer. From the raw replies, several response categories were identified, and each response was then assigned to a category and analysed as an incorrect, neutral or correct statement. Another question on protection against cervical cancer had multiple correct answers and respondents were requested to select all that apply. A total knowledge score was computed by awarding one point per correct answer, with a maximum score of six points.

### Ethical considerations

Ethical clearance to conduct this study was obtained from the University of Pretoria, Faculty of Health Sciences Research Ethics Committee (No. 330/2018) and Stellenbosch University, Health Research Ethics Committee (No. N21/04/040_RECIP_UP_330/2018).

## Results

### Sociodemographic characteristics

A total of 825 potential participants were invited to be part of the present study. Ultimately, 111 participants completed the study questionnaire, of which 55.9% (*n* = 62/111) were from the Western Cape and 44.1% (*n* = 49/111) were from Gauteng. The average age was 19.4 years (16–22 years; median 20 years). The majority, 67.6% (*n* = 75/111), had an education level of Grade 12, while 17.1% (*n* = 19/111) completed Grade 11, 9.0% (*n* = 10/111) completed Grade 10 and the remaining 6.3% (*n* = 7/111) varied between completion of Grade 9, Grade 8 and primary school.

Most of the participants, 73.9% (*n* = 82/111), were living with either their parent(s) or guardian(s), 11.7% (*n* = 13/111) with their partner(s), 5.4% (6/111) with siblings or relatives and another 5.4% (6/111) with spouses. Only 3.6% (*n* = 4/111) either lived alone or with roommates.

**FIGURE 1:**
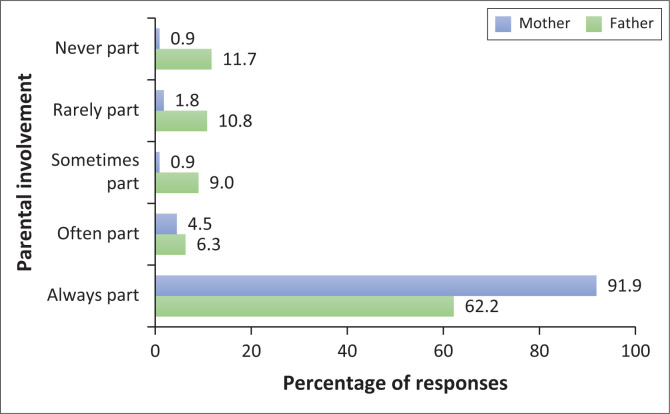
Reported involvement of parents during the teenage years.

The majority of participants, 91.9% (*n* = 102/111), stated that their mothers were always part of their teenage years, while fathers were less frequently involved, with 11.7% (*n* = 13/111) indicating that fathers were never involved during teenage years. Figure 1 shows the responses on parental involvement.

Less than half of the participants (48.6%, *n* = 54/111) have ever been tested for human immunodeficiency virus (HIV), of which 38.9% (*n* = 21/54) were tested in the previous 6 months and 3.7% (*n* = 2/54) tested HIV-positive.

### Self-reported sexual behaviour

Some participants did not respond to all questions in this section, with reasons not known. Most participants, 66.7% (*n* = 74/110), reported having been sexually active in the previous year. Less than one-third of the participants, 28.4% (*n* = 31/109), reported never engaging in a sexual act. The majority of participants who have ever been sexually active, 96.2% (75/78), stated their first time of sexual engagement was in their secondary school years, 0.9% (*n* = 1/78) in their primary school years and the remaining 1.8% (*n* = 2/78) after they had left secondary school.

Among participants who were sexually active, the majority, 60.5% (*n* = 46/76), reported having had one or two partners in total; 91.1% (*n* = 72/79) had male partners; 62.0% (*n* = 49/79) never had sexual intercourse while under the influence of alcohol or drugs, while the majority, 77.2% (*n* = 61/79), used some form of contraception the last time they were sexually active. Table 1 shows the response to questions of sexual behaviour and the use of contraception.

**TABLE 1:** Responses to questions on sexual behaviour and contraception use from participants who reported being sexually active.

Question and responses	Responses	
	*n*	%
**Sexual behaviour**		
‘How many partners have you had in total?’ (*n* = 76)		
1–2	46	60.5
3–5	25	32.9
6–9	3	3.9
> 10	2	2.6
‘My partner(s) are’ (n = 79)		
Male	72	91.1
Female	2	2.5
Both male and female	5	6.3
‘Have you ever had sexual intercourse while under the influence of alcohol or drugs?’ (*n* = 79)		
Always (100% of the time)	0	-
Often (75% of the time)	0	-
Sometimes (50% of the time)	11	13.9
Rarely (25% of the time)	19	24.1
Never under the influence	49	62.0
**Contraception and barrier methods**		
‘During the last time you were sexually active, did you use a form of contraception?’ (*n* = 79)		
Yes	61	77.2
No	18	22.8
‘During the last time you had sexual intercourse, what form(s) of contraception did you use?’ (*n* = 79)		
Hormonal birth control (injectable, the pill, the implant, patch)	20	25.3
Nonhormonal birth control (intrauterine device [IUD])	3	3.8
Condom	26	32.9
Hormonal + condom	6	7.6
Withdrawal	5	6.3
Another barrier method	1	1.3
None	18	22.8
Use of male condoms during the previous 12 months (*n* = 78)		
Never	27	34.6
25% of the time	9	11.5
50% of the time	5	6.4
75% of the time	12	15.4
100% of the time	25	32.1
Use of female condoms during the previous 12 months (*n* = 78)		
Never	73	93.6
25% of the time	1	1.3
50% of the time	0	-
75% of the time	0	-
100% of the time	4	5.1

### Knowledge of cancer of the cervix

Table 2 shows the response categories to the open question ‘Please explain what you understand about cervical cancer (cancer of the mouth of the womb)’. Categories were classified as incorrect, neutral or correct descriptions.

**TABLE 2:** Response to open question on cervical cancer: Number and classified according to correctness.

Response categories to open question: ‘Please explain what you understand about cervical cancer (cancer of the mouth of the womb)’ (*N* = 111)	Incorrect		Neutral		Correct	
	*n*	%	*n*	%	*n*	%
‘Don’t know’/‘Nothing’/No response	-	-	29	26.1	-	-
‘Can prevent you from having children/falling pregnant’	-	-	-	-	16	14.4
‘Cancer of the mouth of the womb/cervix’	-	-	-	-	15	13.5
‘Cancer of private parts/affects only women/cancer like breast cancer’	-	-	-	-	12	10.8
‘Cancer/illness/growth/sores/bleeding of the womb’	-	-	-	-	8	7.2
‘Affects vagina/ovaries’	6	5.4	-	-	-	-
‘STD/Can get from unsafe sex’	-	-	-	-	5	4.5
‘HPV/Virus’	-	-	-	-	4	3.6
‘Dangerous/fatal/incurable disease/silent killer’	-	-	-	-	4	3.6
‘Can get it from family members’/‘Genetically inherited’	4	3.6	-	-	-	-
‘Must have Pap smears/screening/tests’	-	-	-	-	2	1.8
‘Cancer that spreads & infection picked up from toilet/unhealthy lifestyle’	2	1.8	-	-	-	-
‘Your mouth is filled with sores, discomfort in eating/cancer in your mouth’	-	-	2	1.8	-	-
‘Family member has it’	-	-	1	0.9	-	-
‘Prevents you from catching HIV, etc.’	1	0.9	-	-	-	-

HPV, human papillomavirus; HIV, human immunodeficiency virus; STD, sexually transmitted disease.

**FIGURE 2:**
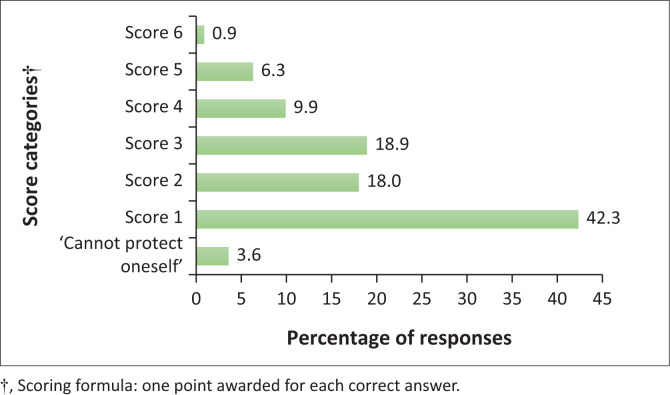
Scored responses to the question on how a woman can protect herself against cervical cancer.

When asked how a woman can protect herself against cervical cancer, the majority, 79.3% (*n* = 88/111), of participants indicated that one must regularly visit the doctor or gynaecologist. Less than half of the participants knew that one must have a regular Pap smear (49.5%, *n* = 55/111), that vaccination can protect one (44.1%, *n* = 49/111) and that condom use can act as protection (20.7%, *n* = 23/111). A small percentage of participants, 13.5% (*n* = 15/111), knew that there are other screening tests available or that ‘having no sex’ (4.5%, *n* = 5/111) can protect one. This question had multiple correct answers, and the results of assigning scores (awarding one point for each correct answer to a maximum score of 6) are shown in Figure 2.

The majority of participants, 80.2% (*n* = 89/111), answered that they have never had a test for cervical cancer. A high number of participants, 70.3% (*n* = 78/111), said that they have heard of a vaccine/injection to prevent cervical cancer. The question ‘did you have a vaccine/injection to prevent cervical cancer’ was answered affirmative by 74.8% (*n* = 83/111) participants, while according to our vaccination records of the VACCS 1 and VACCS 2 projects, 91.9% (*n* = 102/111) of the current participants had been vaccinated.

## Discussion

### Self-reported sexual behaviour

The responses from participants in this study indicated that the majority of young women who have ever been sexually active stated their first time of sexual engagement was in their secondary school years. This finding is in line with the median age of first sexual debut in South African women, which ranges from 16 to 18 years.^19^ The need for primary school immunisation programmes is hereby reiterated because the HPV vaccine is most effective before exposure to HPV^3,4,5^ and allows for increased chance for catch-up vaccination before sexual debut.^20,21,22^ Rebolj et al. conducted an observational study within the English HPV primary screening pilot, exploring the effects of catch-up bivalent HPV vaccination on cervical screening outcomes. Their findings indicate the effectiveness of population-based catch-up campaigns for bivalent HPV vaccination in England. Women were vaccinated aged 24–25 years and were compared with unvaccinated women aged 26–29 years. The detection of HPV 16 and 18 decreased from 3% to 1% (*p* < 0.001), along with a decrease in cervical intraepithelial neoplasia grade 2 (CIN 2) from 6% to 3% (*p* < 0.001), indicating the success of the catch-up campaign.^23^

Although most participants used some form of contraception during the last sexual intercourse, there were poor levels of consistent barrier contraceptive use among those who are sexually active. Such poor utilisation is especially concerning given the high HIV, HPV and sexually transmitted infection (STI) prevalence among young South African women.^24,25^ Furthermore, Menezes et al. reported a high prevalence (47%) of two or more concurrent STIs, specifically chlamydia, gonorrhoea, syphilis, herpes simplex virus type 2 (HSV-2) and high-risk HPV types in young HIV-negative Western Cape South African women.^26^

Encouragingly, the majority of participants (62.0%) reported never having sexual intercourse under the influence of alcohol or drugs. This finding is significant considering research by Mbulawa et al., which demonstrated a clear association between alcohol consumption and increased risk of HPV infection among unvaccinated female learners. Specifically, Mbulawa et al. found that participants who reported ever consuming alcohol had a significantly higher risk of HPV infection (odds ration [OR]: 2.91, 95% confidence interval [CI]: 1.38–6.11, *p* = 0.005).^27^ This association may signify high-risk sexual behaviour, underscoring the importance of promoting responsible sexual practices among young individuals. These findings underscore the need for comprehensive public health strategies that address both behavioural risk factors, such as alcohol consumption, and HPV vaccination, to effectively combat cervical cancer and mitigate HPV-related morbidity and mortality.

### Knowledge of cancer of the cervix and prevention

While most participants understand the importance of regular doctor and/or gynaecologist visits, the results of this study highlighted suboptimal knowledge on the primary prevention of cervical cancer with only 44.1% of respondents knowing that vaccination against HPV and ‘having no sex’ (4.5%) can reduce one’s risk of cervical cancer. Similarly, a poor level of knowledge surrounding the secondary prevention of cervical cancer was displayed with only 49.5% of respondents knowing the importance of regular Pap smears and 13.5% knowing about the availability of other screening tests. Similarly, a study conducted in Northwest Ethiopia assessed the level of comprehensive knowledge regarding cervical cancer among 633 women aged 15 years and older. The findings revealed that approximately 47.5% of the respondents were unsure about the existence of risk factors for cervical cancer, while 17 individuals (2.7%) believed there were no risk factors associated with cervical cancer.^28^ A cross-sectional study conducted in Zimbabwe surveyed 156 women aged 15–50 years, showing a high level of awareness about preventing cervical cancer through HPV vaccination. The study found that among women in the age groups of 15–30 years, 31–40 years and 41–50 years, 91%, 80% and 73%, respectively, understood the importance of HPV vaccination. Additionally, most participants believed that cervical cancer could be prevented by avoiding multiple sexual partners (76.3%), delaying early sexual activity (74.4%) and quitting smoking (66.7%).^29^

Previously, the effectivity of health education linked to vaccination to improve maternal knowledge in the short term was demonstrated.^14,15,16^ Here we observed that this health education had limited effect for longer-term knowledge improvement, as even among HPV vaccine recipients who received education about the subject years ago, current knowledge about its efficacy was low. This highlights the significance of ongoing education, utilising multiple educational platforms.

A systematic review conducted by Flood et al. explored the effects of school-based educational programmes targeting middle adolescent populations (ages 15–17 years) on HPV vaccination uptake and knowledge or perceptions of HPV and associated cancers. The review concluded that implementing such educational interventions enhances HPV vaccination uptake, intentions and attitudes among middle adolescents globally. The authors propose that HPV vaccinations should be offered alongside initial educational interventions and annual follow-up sessions for middle adolescents to optimise vaccination uptake. These interventions, which include information about associated cancers, should be integrated as mandatory school-based programmes tailored to the needs of this specific age group and both genders. It is recommended that interventions be developed collaboratively with input from students, teachers and the wider community beyond the school environment.^30^

Several researchers have also found that health education programmes can increase knowledge about cervical cancer and improve cervical cancer screening uptake.^15,31,32^ Furthermore, women who have prior knowledge about cervical screening tend to seek cervical screening services more often compared to those who have no prior knowledge.^33,34,35^

## Conclusion

The current study highlights a concerning gap in knowledge among young women regarding cervical cancer and its associated risk factors, even after receiving health education integrated with vaccination. Given that the majority of respondents reported their sexual debut during their secondary school years, there is a clear need for ongoing education about HPV, its risk factors and the diseases associated with it among this demographic. Raising awareness is crucial in empowering individuals to take preventive actions. Health education campaigns can play a significant role in achieving this. By providing continued education, individuals can make informed decisions about vaccination and adopt behaviours that reduce their risk of HPV-related diseases, thus contributing to the overall reduction of cervical cancer incidence.

## References

[1] Chesson HW, Dunne EF, Hariri S, Markowitz LE. The estimated lifetime probability of acquiring human papillomavirus in the United States. Sex Transm Dis. 2014 Nov 12;41(11):660–664. 10.1097/OLQ.0000000000000193 25299412 PMC6745688

[2] McGraw SL. Update on prevention and screening of cervical cancer. World J Clin Oncol. 2014;5(4):744–752. 10.5306/wjco.v5.i4.744 25302174 PMC4129537

[3] Wright TC, Van Damme P, Schmitt HJ, Meheus A. Chapter 14: HPV vaccine introduction in industrialized countries. Vaccine. 2006 Aug 31;24(Suppl 3):S3/122–131. 10.1016/j.vaccine.2006.05.118 16949999

[4] Hildesheim A, Herrero R, Wacholder S, et al. Effect of human papillomavirus 16/18 L1 virus like particle vaccine among young women with preexisting infection a randomized trial. JAMA. 2007 Aug 15;298(7):743–753. 10.1001/jama.298.7.743 17699008

[5] Villa LL. Introduction of HPV prophylactic vaccines: A new challenge for public health in the 21st century. Rev Bras Epidemiol. 2008 Sep;11(3):516. 10.1590/S1415-790X2008000300019

[6] South African Government: Basic Education on roll-out of human papillomavirus (HPV) vaccine programme [homepage on the Internet]. 2014 [cited 2024 Feb 25]. Available from: https://www.gov.za/news/basic-education-roll-out-human-papillomavirus-hpv-vaccine-programme-11-feb-2014

[7] Delany-Moretlwe S, Kelley KF, James S, et al. Human papillomavirus vaccine introduction in South Africa: Implementation lessons from an evaluation of the national school-based vaccination campaign. Glob Health Sci Pract. 2018 Oct 3;6(3):425–438. 10.9745/GHSP-D-18-00090 30143561 PMC6172125

[8] Villa LL, Costa RL, Petta CA, et al. Prophylactic quadrivalent human papillomavirus (types 6, 11, 16, and 18) L1 virus-like particle vaccine in young women: A randomised double-blind placebo-controlled multicentre phase II efficacy trial. Lancet Oncol. 2005 May;6(5):271–278. 15863374 10.1016/S1470-2045(05)70101-7

[9] Garland SM, Hernandez-Avila M, Wheeler CM, et al. Quadrivalent vaccine against human papillomavirus to prevent anogenital diseases. N Engl J Med. 2007 May 10;356(19):1928–1943. 10.1056/NEJMoa061760 17494926

[10] World Health Organization. Immunization data portal [homepage on the Internet]. [cited 2024 Mar 02]. Available from: https://immunizationdata.who.int/

[11] Kutz JM, Rausche P, Gheit T, Puradiredja DI, Fusco D. Barriers and facilitators of HPV vaccination in sub-saharan Africa: A systematic review. BMC Public Health. 2023 May 26;23(1):974. 10.1186/s12889-023-15842-1 37237329 PMC10214362

[12] Wigle J, Coast E, Watson-Jones D. Human papillomavirus (HPV) vaccine implementation in low and middle-income countries (LMICs): Health system experiences and prospects. Vaccine. 2013 Aug;31(37):3811–3817. 10.1016/j.vaccine.2013.06.016 23777956 PMC3763375

[13] Botha MH, Van Der Merwe FH, Snyman LC, Dreyer G. The vaccine and cervical cancer screen (VACCS) project: Acceptance of human papillomavirus vaccination in a school-based programme in two provinces of South Africa. S Afr Med J. 2015;105(1):40–43. 10.7196/SAMJ.8419 26046162

[14] Dreyer G, Botha MH, Snyman LC, et al. Combining cervical cancer screening for mothers with schoolgirl vaccination during human papillomavirus (HPV) vaccine implementation in South Africa: Results from the VACCS1 and VACCS2 trials. Int J Gynecol Cancer. 2022; 32(5):592–598. 10.1136/ijgc-2021-003079 35078829

[15] Dreyer G, Van Der Merwe FH, Botha MH, et al. School-based human papillomavirus vaccination: An opportunity to increase knowledge about cervical cancer and improve uptake of screening. S Afr Med J. 2015 Nov 1;105(11):912–916. 10.7196/SAMJ.2015.v105i11.9814 26632317

[16] Snyman LC, Dreyer G, Visser C, Botha MH, Van Der Merwe FH. The vaccine and cervical cancer screen project 2 (VACCS 2): Linking cervical cancer screening to a two-dose HPV vaccination schedule in the South-West district of Tshwane, Gauteng, South Africa. S Afr Med J. 2015;105(3):191–194. 10.7196/SAMJ.8888 26294825

[17] Centres for Disease Control and Prevention. State and local Youth Risk Behavior Survey (YRBS) [homepage on the Internet]. 2017 [cited 2018 Jan 06]. Available from: https://www.cdc.gov/healthyyouth/data/yrbs/pdf/2017/2017_yrbs_standard_hs_questionnaire.pdf

[18] Payne CM. Sexual behavior questionnaire: Association between school-based sex education and contraceptive use among sexually active emerging adults [homepage on the Internet]. University of Florida Digital Collections (UFDC); 2009 [cited 2018 Jan 06]. Available from: https://ufdc.ufl.edu/UFE0024723/00001/pdf/1

[19] Makiwane M, Kwizera S. Youth and well-being: A South African case study. Soc Indic Res. 2009;91(2):223–242. 10.1007/s11205-008-9279-7

[20] Baussano I, Lazzarato F, Ronco G, Dillner J, Franceschi S. Benefits of catch-up in vaccination against human papillomavirus in medium- and low-income countries. Int J Cancer. 2013 Oct 15;133(8):1876–1881. 10.1002/ijc.28197 23564420

[21] Silverberg MJ, Leyden WA, Lam JO, et al. Effectiveness of catch-up human papillomavirus vaccination on incident cervical neoplasia in a US health-care setting: A population-based case-control study. Lancet Child Adolesc Health. 2018 Oct;2(10):707–714. 10.1016/S2352-4642(18)30220-7 30236379 PMC6152835

[22] Martellucci CA, Morettini M, Brotherton JML, et al. Impact of a human papillomavirus vaccination program within organized cervical cancer screening: Cohort study. Cancer Epidemiol Biomarkers Prev. 2022 Mar 1;31(3):588–594. 10.1158/1055-9965.EPI-21-0895 35027435

[23] Rebolj M, Pesola F, Mathews C, Mesher D, Soldan K, Kitchener H. The impact of catch-up bivalent human papillomavirus vaccination on cervical screening outcomes: An observational study from the English HPV primary screening pilot. Br J Cancer. 2022 Jul 20;127(2):278–287. 10.1038/s41416-022-01791-w 35347326 PMC9296648

[24] Giuliano AR, Botha MH, Zeier M, et al. High HIV, HPV, and STI prevalence among young Western Cape, South African women: EVRI HIV prevention preparedness trial. J Acquir Immune Defic Syndr. 2015 Feb 1;68(2):227–235. 10.1097/QAI.0000000000000425 25415290 PMC4378717

[25] Ebrahim S, Mndende XK, Kharsany ABM, et al. High burden of human papillomavirus (HPV) infection among young women in KwaZulu-Natal, South Africa. PLoS One. 2016 Jan 1;11(1):e0146603. 10.1371/journal.pone.0146603 26785408 PMC4718633

[26] Menezes LJ, Pokharel U, Sudenga SL, et al. Patterns of prevalent HPV and STI co-infections and associated factors among HIV-negative young Western Cape, South African women: The EVRI trial. Sex Transm Infect. 2018 Feb 1;94(1):55–61. 10.1136/sextrans-2016-053046 28490581 PMC6561095

[27] Mbulawa ZZA, Somdyala NI, Mabunda SA, Williamson AL. High human papillomavirus prevalence among females attending high school in the Eastern Cape Province of South Africa. PLoS One. 2021 Jun 18;16(6):e0253074. 10.1371/journal.pone.0253074 34143816 PMC8213156

[28] Getahun F, Mazengia F, Abuhay M, Birhanu Z. Comprehensive knowledge about cervical cancer is low among women in Northwest Ethiopia. BMC Cancer. 2013 Dec 2;13(1):2. 10.1186/1471-2407-13-2 23282173 PMC3559275

[29] Nyamambi E, Murendo C, Sibanda N, Mazinyane S. Knowledge, attitudes and barriers of cervical cancer screening among women in Chegutu rural district of Zimbabwe. Cogent Soc Sci. 2020 Jan 18;6(1):1766784. 10.1080/23311886.2020.1766784

[30] Flood T, Wilson IM, Prue G, McLaughlin M, Hughes CM. Impact of school-based educational interventions in middle adolescent populations (15–17yrs) on human papillomavirus (HPV) vaccination uptake and perceptions/knowledge of HPV and its associated cancers: A systematic review. Prev Med (Baltim). 2020 Oct;139:106168. 10.1016/j.ypmed.2020.106168 32603795

[31] Ducray JF, Kell CM, Basdav J, Haffejee F. Cervical cancer knowledge and screening uptake by marginalized population of women in inner-city Durban, South Africa: Insights into the need for increased health literacy. Women’s Health. 2021;17:17455065211047141. 10.1177/17455065211047141 PMC8474337 34553644

[32] Zhang M, Sit JWH, Chan DNS, Akingbade O, Chan CWH. Educational interventions to promote cervical cancer screening among rural populations: A systematic review. Int J Environ Res Public Health. 2022;19(11):6874. 10.3390/ijerph19116874 35682457 PMC9180749

[33] Mupepi SC, Sampselle CM, Johnson TRB. Knowledge, attitudes, and demographic factors influencing cervical cancer screening behavior of Zimbabwean women. J Womens Health. 2011 Jun;20(6):943–952. 10.1089/jwh.2010.2062 21671779

[34] Compaore S, Ouedraogo CMR, Koanda S, Haynatzki G, Chamberlain RM, Soliman AS. Barriers to cervical cancer screening in Burkina Faso: Needs for patient and professional education. J Cancer Educ. 2016 Dec 4;31(4):760–766. 10.1007/s13187-015-0898-9 26336956 PMC4779069

[35] Gwavu Z, Murray D, Okafor UB. Perception of women’s knowledge of and attitudes towards cervical cancer and Papanicolaou smear screenings: A qualitative study in South Africa. Healthcare. 2023;11(14):2089. 10.3390/healthcare11142089 37510530 PMC10379022

